# IL-38 has an anti-inflammatory action in psoriasis and its expression correlates with disease severity and therapeutic response to anti-IL-17A treatment

**DOI:** 10.1038/s41419-018-1143-3

**Published:** 2018-10-30

**Authors:** Laura Mercurio, Martina Morelli, Claudia Scarponi, Elan Z. Eisenmesser, Nunzianna Doti, Gianluca Pagnanelli, Emanuela Gubinelli, Cinzia Mazzanti, Andrea Cavani, Menotti Ruvo, Charles A. Dinarello, Cristina Albanesi, Stefania Madonna

**Affiliations:** 1 Laboratory of Experimental Immunology and Integrated Research Center for PSOriasis (CRI-PSO), Istituto Dermopatico dell‘Immacolata IDI-IRCCS, via Monti di Creta, 104, ROME, Italy; 2 Section of Dermatology, Department of Medicine, University of Verona, P.zza Stefani, 1, Verona, 37126 Italy; 3 Department of Biochemistry & Molecular Genetics, School of Medicine, University of Colorado Denver, Anschutz Campus, Aurora, 80045 CO USA; 4 Istituto di Biostrutture e Bioimmagini-CNR and CIRPEB, Via Mezzocannone, 16, Naples, 80134 Italy; 5 1st Division of Dermatology and CRI-PSO, Istituto Dermopatico dell‘Immacolata IDI-IRCCS, via Monti di Creta, 104, Rome, 00167 Italy; 6 CRI-PSO Istituto Dermopatico dell’Immacolata, IDI-IRCCS, via Monti di Creta, 104, Rome, 00167 Italy; 7 INMP/NIHMP, via di S.Gallicano, 25, Rome, 00153 Italy; 8 Department of Medicine, Radboud University Medical Center, 6525 HP Nijmegen, The Netherlands; 9 Department of Medicine, School of Medicine, University of Colorado, Denver, Anschutz Campus, Aurora, CO USA

## Abstract

IL-36 cytokines, a subgroup of IL-1 family, comprise IL-36α, IL-36β, and IL-36γ agonists, abundantly expressed in psoriatic skin, and IL-36RA and IL-38 antagonists. In psoriatic skin, IL-36 cytokines interfere with keratinocyte cornification programs and induce the release of antimicrobial peptides and chemokines active on neutrophils and Th17 lymphocytes. To date, the role of IL-38 antagonist in psoriasis remains to be defined. Here, we demonstrate that skin and circulating IL-38 levels are reduced in psoriatic patients and in other skin diseases characterized by neutrophilic infiltrate. In psoriasis, the balance of IL-36γ agonist/IL-38 antagonist serum levels is in favor of agonists and is closely associated with disease severity. Interestingly, IL-38 is upregulated by anti-IL-17A biological treatment and positively correlates with the therapeutic efficacy of secukinumab in psoriatic patients. The downregulation of IL-38 expression is strictly related to keratinocyte de-differentiation triggered by the inflammatory cytokines IL-36γ, IL-17, and IL-22. Finally, we demonstrate that administration of recombinant full-length IL-38 counteracts in vitro the biological processes induced by IL-36γ in human keratinocytes and endothelial cells and attenuates in vivo the severity of the psoriasiform phenotype induced by IMQ in mice. Such effects are achieved by restoring the physiological programs of keratinocyte proliferation and differentiation, and reducing the immune cell infiltrates.

## Introduction

Psoriasis is an immune-mediated skin disease in which interferon (IFN)-γ, tumor necrosis factor (TNF)-α, interleukin (IL)-17, and IL-22 cytokines, released by Th1 and Th17 lymphocytes^1,2^, have a pathogenic action by promoting hyperproliferation, interfering with the terminal differentiation and inducing the secretion of pro-inflammation molecules by keratinocytes^3,4^. A growing number of studies demonstrated that also IL-36 cytokines are pathogenic drivers of psoriasis^5,6^. IL-36s belong to IL-1 family and comprise three agonists, IL-36α, IL-36β, and IL-36γ, and two receptor antagonists IL-36RA and IL-38^7^. IL-36 agonists are strongly expressed in psoriatic skin of individuals affected by plaque psoriasis and generalized pustular psoriasis. Here, these cytokines have inflammatory effects on many cell targets, mainly keratinocytes, by interfering with their cornification programs and inducing the release of antimicrobial peptides and chemokines active on neutrophils and Th17 lymphocytes^8^. IL-36s also promote proliferation and migration of human dermal microvascular endothelial cell (HDMEC), thus contributing to the dermal capillary dilatation typical of psoriatic lesions^9^. Although human T lymphocytes do not express the IL-36R receptor (IL-36R), IL-36 cytokines indirectly promote Th17 lymphocyte polarization by activating the maturation of dendritic cells^10–12^. IL-17, together with TNF-α and IL-22, upregulates IL-36 themselves leading to a local auto-amplification loop^13^. The role of IL-36 agonists in the pathogenesis of psoriasis has been widely demonstrated. Capon et al. recently showed that IL-36R blockade by IL-36Ra or a neutralizing IL-36R antibody decreases the inflammation in ex vivo and in vivo experimental models of psoriasis^14^. However, the role of IL-36 antagonists, in particular of IL-38, remains yet undefined. Mutations in IL-36Ra have been described as a cause of pustular psoriasis, owing to an impaired inhibitory activity of IL-36Ra on Th17 responses^15–17^. In parallel, IL-38 allelic variants have been correlated to rheumatic diseases, including psoriatic arthritis^18^. IL-38 is a 17–18 kDa protein sharing 40% sequence similarity with IL-1RA and IL-36Ra antagonists and elicits its antagonistic effects through binding to IL-36 receptor, as IL-36Ra^7,17^. IL-38 is dramatically reduced in the epidermis of psoriatic lesions as compared with uninvolved or healthy skin, in line with its reduced expression observed in de-differentiated keratinocytes compared with differentiated cells^19,20^. IL-38 reduction is peculiar of chronic psoriatic skin, as its expression is contrarily induced in synovial tissues of patients with rheumatoid arthritis and in colonic inflamed biopsies of patients with Chron’s disease^19^. Interestingly, IL-38 has anti-inflammatory effects on mouse models of arthritis and on a model of retinopathy, where it suppresses the secretion of chemokines involved in Th17 pathway and inhibits the pathological processes of vascularization, respectively^21,22^.

In this study, we analyzed the potential involvement of IL-38 in psoriasis by evaluating its circulating and skin levels in affected patients before and after the biological inhibition of IL-17A with secukinumab. Furthermore, we investigated the effects of IL-38 administration in both in vitro and in vivo experimental models of psoriasis, such as in human keratinocyte and endothelial cell cultures activated by pro-inflammatory cytokines related to psoriasis, as well as in the IMQ-induced murine model of skin inflammation.

## Results

### Skin levels of IL-38 are reduced in psoriatic patients and in other skin diseases characterized by neutrophilic infiltrate

**Fig. 1:**
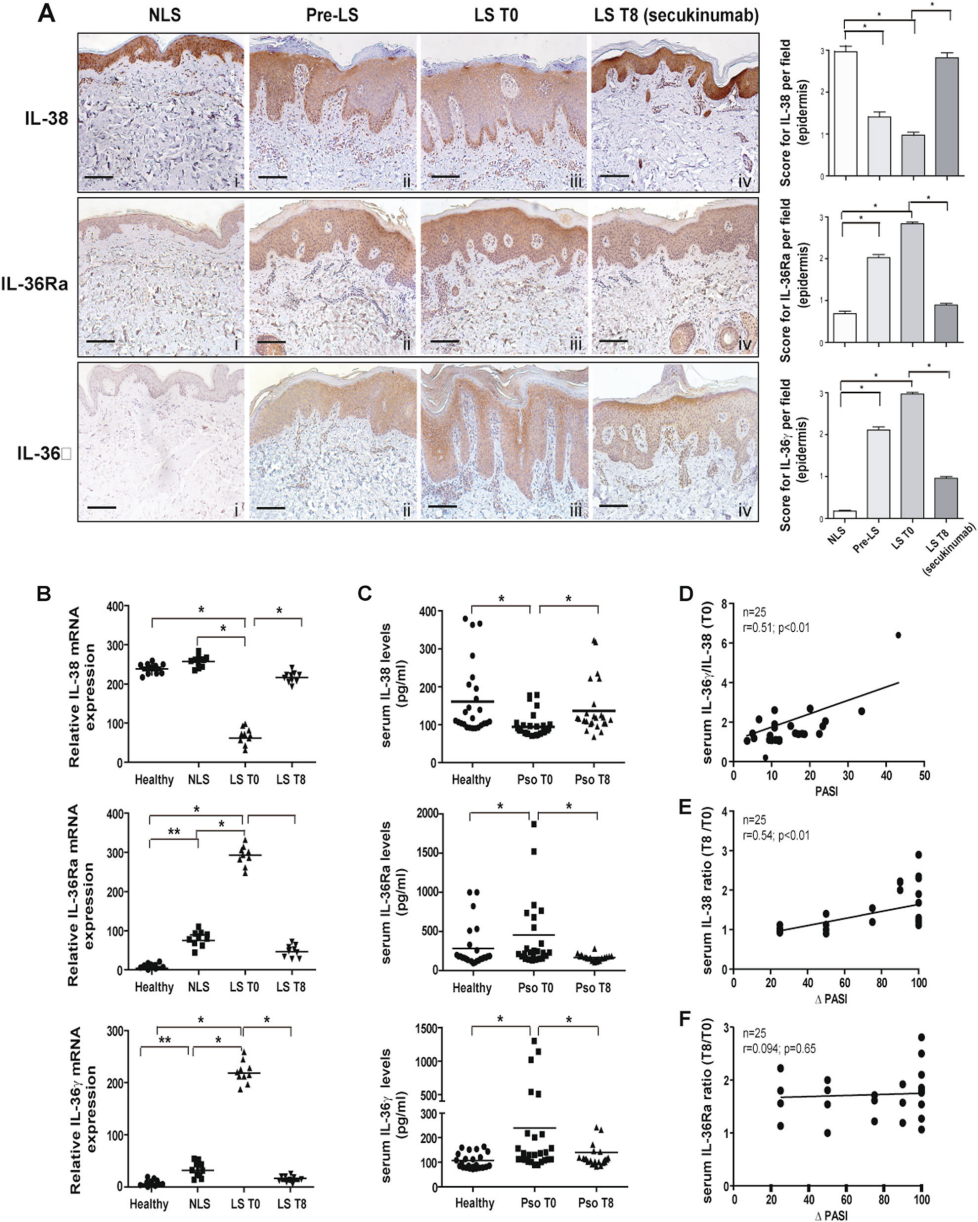
IL-38 levels are reduced in skin and serum of psoriatic patients and upregulated by secukinumab treatment. **a** IHC for IL-38, IL-36Ra, and IL-36γ (all stained in red-brown) was performed on paraffin-embedded sections of biopsies obtained from psoriatic skin (*n* = 8) including not lesional (NLS) (i), proximal-to-lesion (Pre-LS) (ii), lesional zones of evolving plaques, before (LS T0) (iii) and after 8 weeks (LS T8) (iv) of secukinumab treatment. Sections were counterstained with Mayer’s H&E. One out of eight representative stainings of psoriatic skin biopsies are shown. Bars, 100 μm. Graphs show the mean of four-stage score values for IL-38, IL-36Ra, and IL-36γ ± SD epidermal expression per three different fields of all six sections. *p ≤ 0.05, as assessed by Mann–Whitney *U* test. **b** mRNA expression of IL-38, IL-36Ra, and IL-36γ were analyzed by RT-PCR on healthy, NLS, LS T0, and LS T8 biopsies (*n* = 10) and normalized to HPRT-1 levels. **c** Cytokine levels were analyzed by ELISA in serum obtained by venous blood of healthy (*n* = 25) and psoriatic individuals (*n* = 25) before (Pso T0) and after (Pso T8) secukinumab treatment. **b**, **c** The results are shown as individual values and mean. *p ≤ 0.05; p** ≤ 0.01, as assessed by Mann–Whitney *U* test. **d** Correlation between the ratio of IL-36γ and IL-38 serum levels and PASI (psoriasis area and severity index) at baseline. **e**, **f** Correlations between the ratio of IL-38 or IL-36Ra between T8 and T0 and Δ PASI (Δ PASI, calculated as percentage of PASI reduction) score in psoriatic individuals after secukinumab treatment. Spearman’s test was used for correlation analysis. *r* indicates the strength of the linear relationship, *n* indicates the number of values in each plot, and *p* value shows the probability that the slope of the true relationship is zero. *p* ≤ 0.05 was considered significant

Aimed at clarifying the controversial IL-38 expression in psoriatic and healthy skin^19,23^, levels of IL-38, together with IL-36Ra and IL-36γ, were analyzed in psoriatic specimens, including non-lesional (NLS) skin, and skin overlapping pre-lesional (Pre-LS) and lesional (LS T0) zones of target plaques by immunohistochemistry. As shown in Fig. 1a, a strong cytoplasmic expression of IL-38 was observed throughout the epidermis of NLS areas (panel i), as well as of healthy skin (Fig. S1, panel i), whereas a lower immunoreactivity was detected in dermal endothelial cells and fibroblasts. During the transition from Pre-LS to LS T0 area of the same skin biopsy, IL-38 staining progressively decreased in the epidermis (Fig. 1a, panel ii). This reduction was more evident in LS areas, where IL-38 expression was moderately detected in the proliferative layer of epidermis, but it was absent in suprabasal layers (Fig. 1a, panel iii). IL-36Ra and IL-36γ were minimally expressed in NLS (Fig. 1a, panels i) and healthy specimens (Fig. S1, panel i), whereas their levels were enhanced in the upper layers of pre-LS and LS T0 epidermis (Fig. 1a, panels ii and iii). Infiltrating immune cells were also positive for IL-36γ and IL-38 in pre-LS and LS T0 skin, in accordance with IL-36γ staining previously detected in macrophages, dendritic cells, and Langerhans cells^24^ (mean percentage of IL-36γ^+^ cells: 10 ± 1.2% and 38 ± 1.4% in pre-LS and LS T0 skin, respectively; IL-38^+^ cells: 15 ± 1.1% and 35 ± 1.9% in pre-LS and LS T0 skin, respectively, Fig. 1a, panels ii and iii). Contrarily, immune cells infiltrating psoriatic dermis were quite negative for IL-36Ra (Fig. 1a, panels ii and iii). IL-38 epidermal downregulation was observed in other inflammatory skin diseases, characterized by a diffuse neutrophilic infiltrate, where modest levels of IL-38 were observed only in the upper layers of the epidermis (Fig. S1, panels ii, iii, iv). Vice versa, a higher, but variable, immunoreactivity for IL-38 was detected throughout the epidermis of lesional AD (Fig. S1, panel v). Differently from IL-38, IL-36Ra, and IL-36γ were upregulated in the epidermis of Hidradenitis suppurativa (HS), Sweet Syndrome (SS), Pyoderma Gangrenosum (PG), and Atopic Dermatitis (AD), as compared with healthy skin, with different localization among disorders. In particular, IL-36Ra and IL-36γ expression was diffuse throughout the epidermis of PG and AD (Fig. S1, panels iv and v), whereas it was localized in the upper and lower layers of HS and SS epidermis, respectively (panels ii and iii). In all diseased skin biopsies, a modest IL-38 expression was observed in immune cells infiltrating the dermis (Fig. S1, panels i–v). Here, with the exception of AD, a relevant percentage of IL-36Ra^+^ and IL-36γ^+^ cells with a neutrophil-like morphology was observed in immune cells.

### IL-38 is upregulated by anti-IL-17A treatment and correlates to the therapeutic efficacy to secukinumab

To evaluate the effects of anti-IL-17A treatment on IL-38, its expression was analyzed in vivo in skin biopsies taken 8 weeks after therapy with secukinumab from the same target plaques analyzed at baseline. As shown in Fig. 1a (panel iv), IL-38 positivity increased in skin of secukinumab-treated patients (mean of LS T8: 2.8 ± 0.3) as compared with levels before treatment (mean of LS T0: 1.0 ± 0.2, *p* ≤ 0.05), becoming similar to those observed in NLS or healthy skin (mean of NLS: 3.0 ± 0.3) Fig. 1a and Fig. S1, respectively, panels i). Contrarily, IL-36Ra and IL-36γ staining was strongly downregulated (IL-36Ra: 0.9 ± 0.3 in LS T8 vs 2.9 ± 0.1 in LS, *p* ≤ 0.05; IL-36γ: 1.0 ± 0.2 in LS T8 vs 3.0 ± 0.1 in LS, *p* ≤ 0.05), according to previous observations (Fig. 1a, panels iv)^24,25^. IL-38 mRNA levels also increased in skin after 8-week therapy, compared with those detected before treatment (means: 215.0 in LS T8 vs 82.5 in LS T0, *p* ≤ 0.05), whereas IL-36Ra and IL-36γ mRNA was reduced (IL-36Ra: 295.0 in LS T8 *vs* 52.5 in LS T0, *p* ≤ 0.05; IL-36γ: 215.0 in LS T8 vs 15.5 in LS T0, *p* ≤ 0.05) (Fig. 1b). Next, we investigated the potential systemic relevance of IL-38 by measuring its levels in serum of psoriatic patients before and after secukinumab. At baseline, IL-38 levels were significantly lower in psoriatic patients than in healthy donors, and were upregulated following secukinumab (155.0 in Pso T8 vs 95.0 in Pso T0, *p* ≤ 0.05) (Fig. 1c). IL-36Ra and IL-36γ serum release was instead increased at baseline of the same psoriatic patients, compared with healthy group (means of IL-36Ra: 490.0 in Pso T0 vs 355.0 in healthy, *p* ≤ 0.05; IL-36γ: 255.0 in Pso T0 *vs* 115.0 in healthy, *p* ≤ 0.05), and reduced by anti-IL-17A therapy (means of IL-36Ra: 490.0 in Pso T0 vs 150.0 in Pso T8, *p* ≤ 0.05; IL-36γ: 255.0 in Pso T0 vs 135.0 in Pso T8, *p* ≤ 0.05) (Fig. 1c). As shown in Fig. 1d, the ratio of serum IL-36γ/IL-38 levels in psoriatic patients strongly correlated to the disease activity as determined by psoriasis area and severity index (PASI) scores (*r* = 0.51, *p* < 0.01). The ratio of serum levels of IL-38, but not of IL-36Ra, after and before secukinumab resulted to be closely associated with the resolution of the clinical psoriatic manifestations (*r* = 0.51, *p* < 0.01, and *i* = 0.094, *p* = 0.65, respectively), evaluated as percentage of PASI reduction determined after and before treatment (Fig. 1e, f).

### IL-38 and IL-36Ra expression is differently regulated in human keratinocytes

**Fig. 2:**
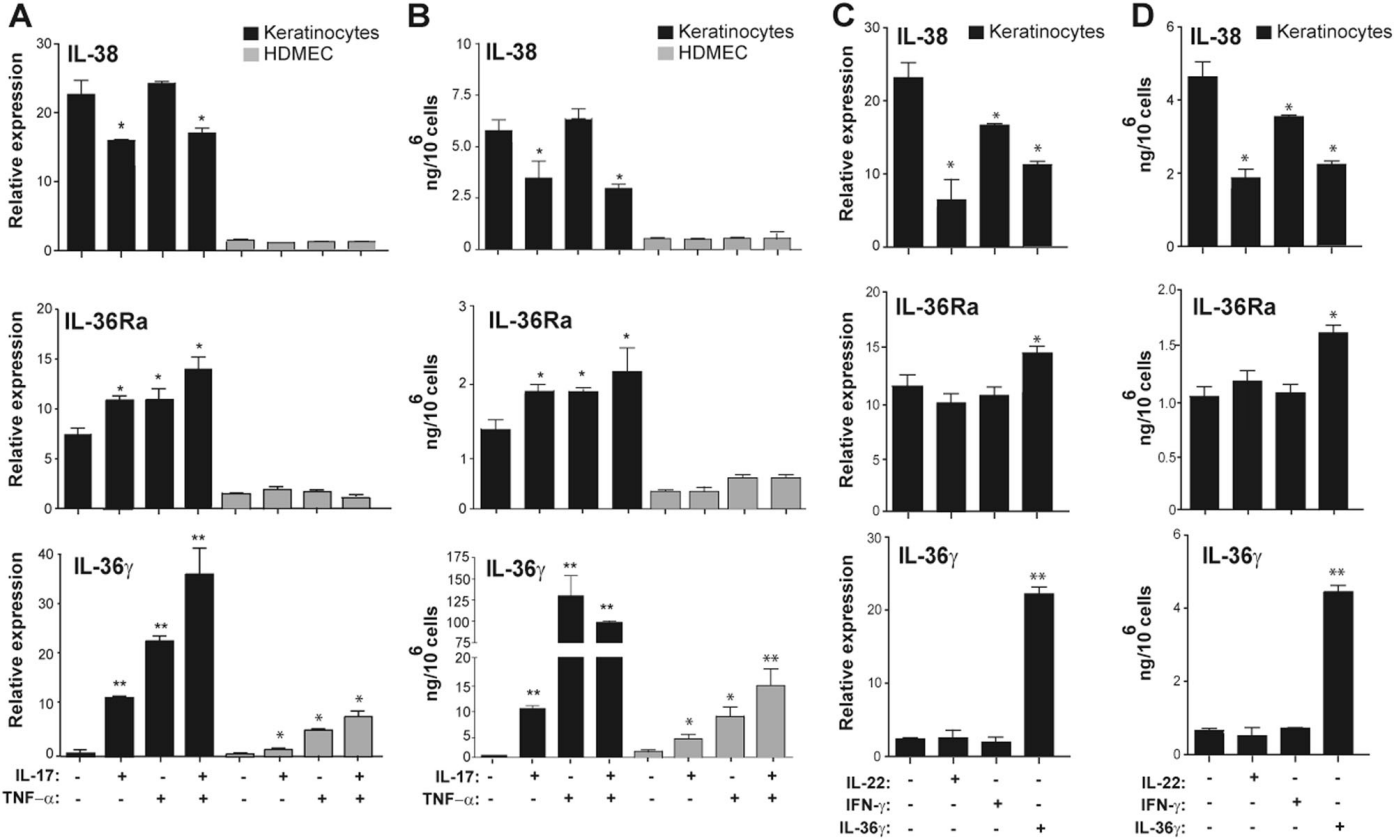
Regulation of the expression and release of IL-38, IL-36Ra, and IL-36γ cytokines by psoriasis-related cytokines in human keratinocytes and endothelial cells. **a**, **c** IL-38, IL-36Ra, and IL-36γ mRNA expression was detected by real-time PCR in keratinocyte cultures undergoing terminal differentiation (4 days post confluency) and HDMEC cultures stimulated with IL-17A (50 ng/ml) and TNF-α (50 ng/ml for keratinocytes and 10 ng/ml for HDMEC), alone or in combination **a** or in keratinocytes stimulated with IL-22 (50 ng/ml), IFN-γ (200 U/ml, or IL-36γ (50 ng/ml) **c**, for 6 h. GAPDH mRNA levels were detected for normalization. **b**, **d** IL-36 cytokine levels were analyzed by ELISA in supernatants obtained by cell cultures after 24 h cytokine stimulation. All data shown are the mean of three different experiments. *p ≤ 0.01 and **p ≤ 0.001 compared with untreated cultures, as assessed by Mann–Whitney *U* test

As IL-36 cytokines inhibit keratinocyte terminal differentiation during psoriasis development^26^, we investigated IL-38 expression in post-confluent keratinocyte cultures, resembling differentiated cells of the epidermal suprabasal layer^11,27^. Keratinocytes, but not HDMEC, constitutively expressed and released IL-38 and, at lower extent, IL-36Ra (Fig. 2a, b). IL-38 and IL-36Ra were also expressed in sub-confluent (proliferating) keratinocyte cultures in basal condition, even though at reduced levels as compared with differentiated cultures (approximately fivefolds lower, data not shown). mRNA expression and release of IL-38 were significantly downregulated by IL-17A in post-confluent keratinocyte cultures, whereas they were not influenced by TNF-α (Fig. 2a, b). In parallel, IL-17A and TNF-α, alone and, at higher extent in combination, significantly induced IL-36γ and IL-36Ra (Fig. 2a, b). mRNA and protein levels of IL-36γ were also significantly induced in HDMECs, but at a lower extent compared with keratinocytes (Fig. 2a, b). Interestingly, IL-38 levels were also strongly downregulated by IL-22 and, at a lower extent, by IFN-γ and IL-36γ in keratinocytes (Fig. 2c, d). Vice versa, in line with literature data^28^, we found that IL-36γ was induced by IL-36γ itself, whereas it was not influenced by IFN-γ and IL-22 (Fig. 2c, d). IL-36Ra was also upregulated in keratinocytes by IL-36γ, but not influenced by IFN-γ and IL-22 (Fig. 2c, d). As a whole, these data demonstrate that keratinocytes are the main producers of IL-38 in resident skin cells, and its expression is downregulated by inflammatory cytokines.

### IL-38 counteracts the biological processes induced by IL-36γ in human keratinocytes and dermal vascular endothelial cells

**Fig. 3:**
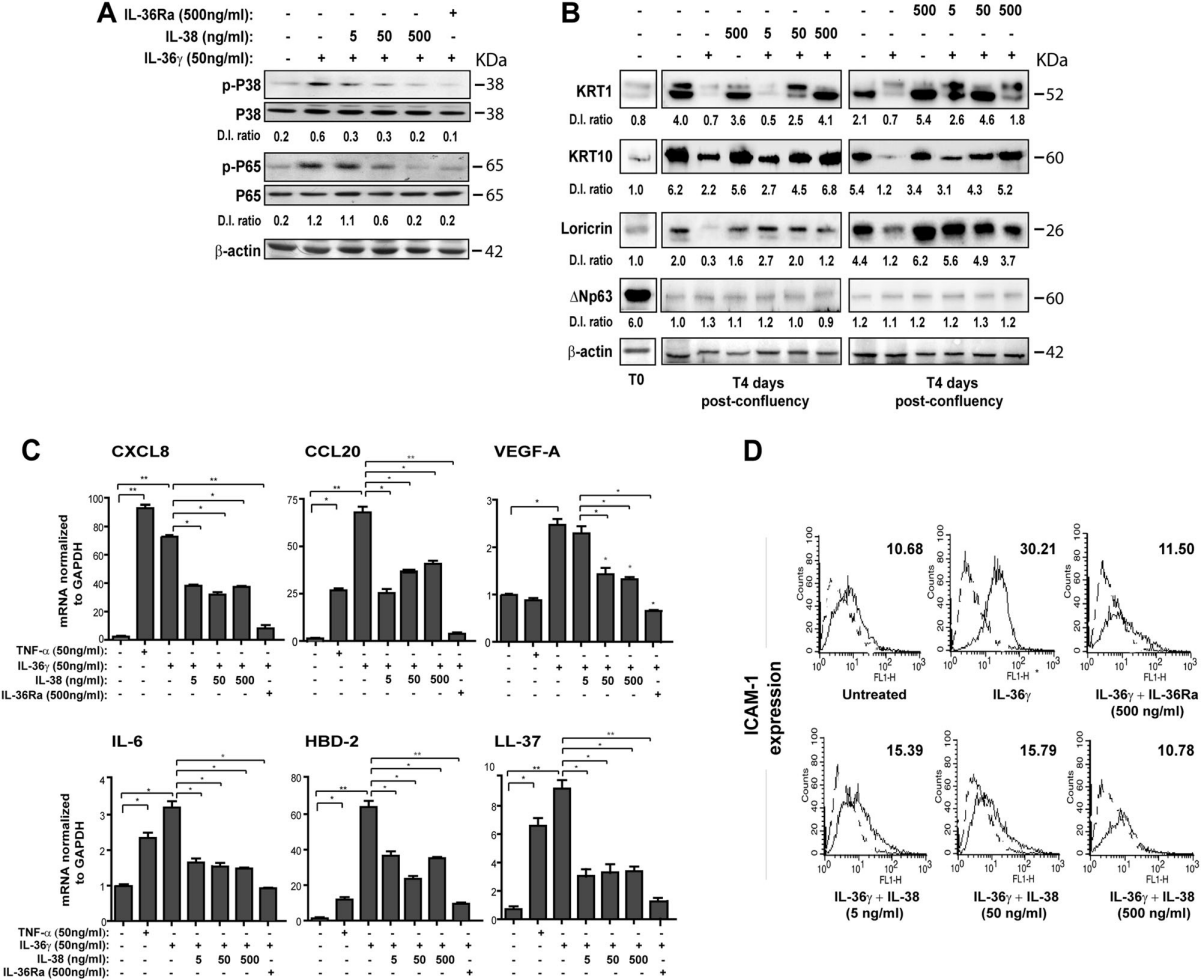
IL-38 inhibits IL-36γ-induced P38 and NF-κB signalings, as well as regulates the expression of differentiation markers and inflammatory molecules in human keratinocytes. All experiments were performed on keratinocyte cultures (*n* = 3 strains) undergoing terminal differentiation and stimulated or not with IL-36γ in presence or absence of the indicated doses of IL-38 or IL-36Ra. **a** Protein extracts were obtained from keratinocyte cultures stimulated for 24 h and subjected to WB analysis to detect P38 and P65 phosphorylation. Filters were probed with anti-P38 and -P65 Abs. b Keratinocyte cultures were analyzed for KRT1, KRT10, Loricrin and ΔNp63 expression by WB. **a**, **b** β-actin was used as loading control and DI ratio indicates the densitometric intensity of the indicated phosphorylated/unphosphorylated proteins shown in one representative of three different WB. **c** mRNA levels of CXCL8, CCL20, VEGF-A, IL-6, HBD-2, and LL-37 were detected by real-time PCR analysis in keratinocytes stimulated for 6 h and normalized for GAPDH mRNA levels. TNF-α treatment was used as positive control. **d** ICAM-1 expression was evaluated by flow cytometry analysis on keratinocytes stimulated for 24 h with IL-36γ (50 ng/ml) and shown as mean fluorescence intensity. All data shown are the mean of three different experiments. **c** *p ≤ 0.05 and p** ≤ 0.01 compared with untreated or IL-36γ-treated cultures, as assessed by Mann–Whitney *U* test

As shown in Fig. 3a, similarly to IL-36Ra^7,29^, IL-38 inhibited the phosphorylation of P38 MAPK and the subunit P65 of NFκB induced by IL-36γ (Fig. 3a). Other than inducing the expression of chemokines attracting neutrophils and T cells, IL-36 cytokines interfere with keratinocyte differentiation in organotypic models by downregulating filaggrin^8,13^. We thus analyzed the effects of IL-38 on differentiation and inflammatory responses induced by IL-36γ in keratinocytes. As shown in Fig. 3b, post-confluent keratinocyte cultures expressed higher levels of differentiation markers, such as KRT1 and KRT10 and loricrin, compared with proliferating cells (T0), and their expression was abrogated by IL-36γ. Interestingly, IL-38 dose-responsively upregulated and normalized the levels of KRT1, KRT10, and loricrin, but not those of ΔNp63, a typical marker of proliferating keratinocytes. Similarly, IL-36Ra increased KRT1, KRT10, and loricrin levels in IL-36γ-treated keratinocytes, although these effects were more evident at lower doses, compared with IL-38 (Fig. 3b). IL-38 also reduced the IL-36γ-induced expression of CXCL8, CXCL20, IL-6, and vascular endothelial growth factor A (VEGF*-A*), as well as of antimicrobial peptides HBD-2 and LL-37, although a dose–response was not observed (Fig. 3c). At high doses, IL-36Ra counteracted the expression of most inflammatory mediators more efficiently than IL-38 (Fig. 3c). IL-38 also downregulated the IL-36γ-induced expression of intercellular adhesion molecule 1 (ICAM-1), with a significant reduction at higher dose (Fig. 3d). HDMEC also express IL-36 receptor and, upon IL-36γ, they express pro-inflammatory chemokines and adhesion molecules, whose levels are reduced by IL-36Ra^30^. IL-36γ not only synergizes with TNF-α in the release of these inflammatory mediators, but it induces proliferation of cultured HDMEC. As shown in Fig. S2, IL-38 inhibited the phosphorylation of ERK1/2 strongly induced by IL-36γ in HDMEC, as well as that of P65 (panel a). Interestingly, HDMEC proliferation induced by IL-36γ was significantly attenuated by IL-38 (Fig. S2b). IL-38, similarly to IL-36Ra, also reverted the IL-36γ- and TNF-α-induced expression of inflammatory molecules in HDMEC cultures (Fig. S2b–d).

### IL-38 ameliorates the psoriasiform phenotype of IMQ-treated mice

**Fig. 4:**
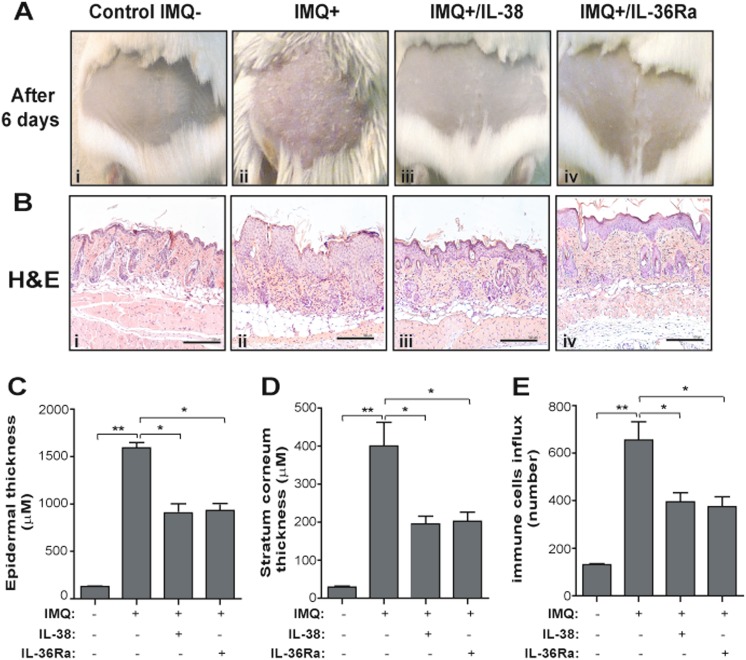
Ameliorative effects of IL-38 administration on pathological changes of IMQ-induced psoriasiform murine skin lesions. **a** Macroscopic views of back skin from mice left untreated (IMQ-) (i), IMQ-treated (IMQ + ) (ii) or co-treated with IMQ and IL-38 (IMQ + /IL-38) (iii) or IMQ and IL-36Ra (IMQ + /IL-36Ra) (iv), both subcutaneously injected, after 5 days. **b** Representative H&E staining of untreated (i), treated with IMQ cream (ii), in presence of IL-38 (iii) or IL-36Ra (iv). Bars, 500 μm. Subcutaneous injection of IL-38 or IL-36Ra reverted the phenotypic skin changes determined by IMQ. The quantifications of epidermal **c**, scale thickness **d**, and immune cell influx **e** were analyzed as parameters of skin acanthosis and inflammation. Graphs show means of microns of epidermis and stratum corneum thickness, and mean of number of infiltrating immune cells per section (*n* *=* 3 sections) ± S.D. per group (*n* = 8 mice). p* ≤ 0.01 and **p ≤ 0.001 compared with untreated or IMQ-treated groups, as assessed by unpaired Student‘s *t* test

**Fig. 5:**
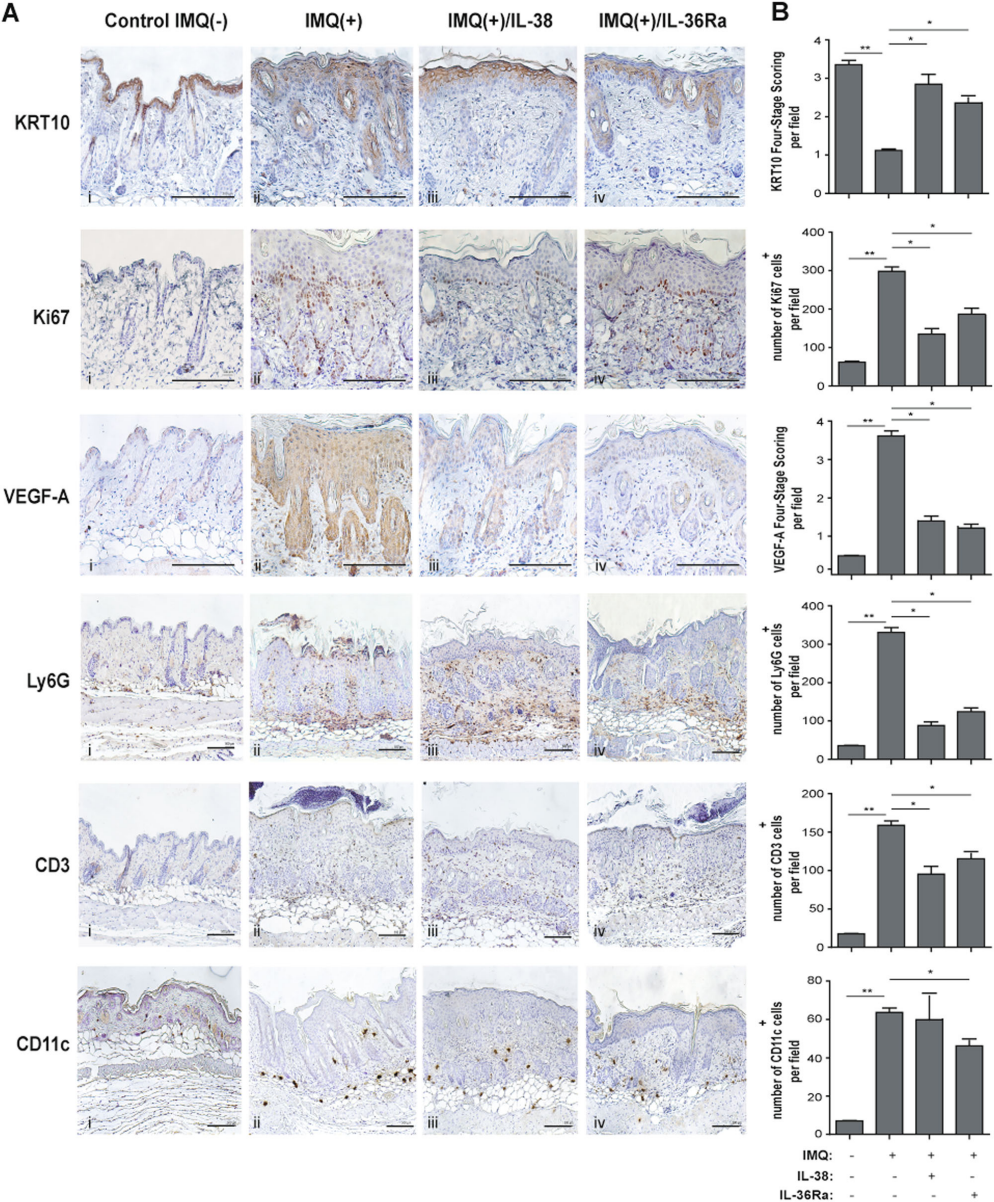
IL-38 reverts the pathological markers in IMQ-induced murine model of psoriasis. **a** (i), IMQ-treated (*n* = 8) (ii) and co-treated with IMQ and IL-38 (IMQ + /IL-38) (*n* = 8) (iii) or IL-36Ra (IMQ + /IL-36Ra) (*n* = 8) (iv) shows an upregulated and normalized expression of KRT10 in the epidermis after IL-38 and IL-36Ra treatment, as well as a reduction of VEGF-A expression, and of positive Ki67, Ly6G, CD3, and CD11c cells. Sections were counterstained with Mayer’s H&E and were visually evaluated by a pathologist experienced in dermatology. One out of eight representative stainings is shown. Bars, 500 μm. **b** Graphs show the mean of number of positive cells or of four-stage score values for KRT10 ± SD per three sections per experimental group (six of eight mice were analyzed). *p ≤ 0.01, **p ≤ 0.001 compared with untreated or IMQ-treated groups, as assessed by unpaired Student‘s *t* test

We further investigated IL-38 potential in IMQ-induced psoriasiform dermatitis, an IL-17/IL-22-mediated mouse model of the disease^31^. Subcutaneous injection of IL-38 for 5 days, concomitantly to topical IMQ application, ameliorated the psoriasiform manifestations, with a ~ 40% reduction of acanthosis and consequent scale thickness, as well as of the dermal inflammatory infiltrate (~ 50% decrease) (Fig. 4a–e). As shown in Fig. 5, epidermal differentiation markers, such as KRT10, which are normally expressed in the suprabasal layers of epidermis, displayed a broad staining throughout keratinocyte layers of IMQ-induced skin lesions (Fig. 5a, b). Interestingly, administration of IL-38 normalized the expression of KRT10, which compartmentalized to the upper granular layers, as typically observed in normal epidermis (Fig. 5a). A reduced number of proliferating Ki67^+^ keratinocytes was also observed in IL-38-treated inflamed skin (Fig. 5a, b). In addition, the epidermis of IL-38-treated mice showed a reduced expression of the pro-angiogenic VEGF-A cytokine, compared with controls (Fig. 5a, b). IL-38 treatment also decreased the number of infiltrating CD3^+^ T lymphocytes and Ly6G^+^ neutrophils (panels iii), compared with IMQ-treated mice group (panels ii), whereas it did not influence the number of CD11c^+^ cells (panels ii and iii). Both IL-36Ra and IL-38 administration determined a significant reduction of the endogenous levels of IL-36Ra (~ 30% decrease of relative mRNA expression) and IL-36γ (~ 35% decrease), whereas they did not influence endogenous IL-38 expression (data not shown). In parallel, also CCL20, CXCL8, and IL-6 mRNA expression was downregulated in the skin of IL-38-treated mice group (data not shown). Finally, similarly to IL-38, IL-36Ra ameliorated the psoriasiform phenotype, with positive effects on all pathological markers analyzed (Fig. 5, panels iv).

## Discussion

In this study, we identify IL-38 as a new responsive biomarker for psoriasis, and demonstrate its anti-inflammatory properties in in vitro and in vivo experimental models of psoriasis. The interest toward IL-38 in psoriatic context arises from our and earlier observations that its expression is reduced in the epidermis of skin lesions and in the serum of psoriatic patients, as compared with uninvolved or healthy skin, and is inverse to those of IL-36Ra, a well-characterized antagonist of IL-36 cytokines strongly induced in psoriatic lesions^32^. We found that the balance of IL-36γ agonist/IL-38 antagonist levels, but not of IL-36γ/IL-36Ra, in serum of psoriatic patients is closely associated with disease severity. Interestingly, IL-38 expression is responsive to IL-17A inhibition, as its levels significantly increase in skin and in serum of affected patients after treatment with secukinumab, a selective anti-IL-17A antibody used in clinical practise^33^, whereas IL-36γ and IL-36Ra levels decrease in line with previous studies^24,25^. We show that the ratio of IL-38 serum levels after and before secukinumab correlates to the therapeutic efficacy in psoriatic patients, thus identifying IL-38 as a potential responsive biomarker for psoriasis. However, these findings need to be further confirmed on a larger cohort of psoriatic patients treated by secukinumab and other biologicals, such as antibodies blocking TNF-α, a strong inducer of IL-36 agonists. Taken together, our findings unveil a relevant role of IL-38 in psoriasis, supporting the hypothesis that the dysregulation of its expression could be pathogenic in psoriasis. IL-38 and IL-36Ra antagonists have anti-inflammatory properties by antagonizing the IL-36 pathways through a direct binding to IL-36 receptor with similar affinities^5,7^. However, the upregulation of IL-36Ra alone might be not sufficient to unleash IL-36-dependent inflammatory responses in psoriatic lesions, where several cytokines inducing IL-36 are active on resident skin and immune cells. Owing to the different in vitro and in vivo expression of IL-38 and IL-36Ra, IL-38, and IL-36Ra can be considered as not redundant cytokines, having IL-38 additional functions closely related to the keratinocyte differentiation. In fact, the loss of IL-38 in the suprabasal layers of psoriatic epidermis is associated with the impaired differentiation processes occurring in the affected skin, to which IL-17 and IL-22 cytokines markedly contribute^1,34^. In support of this hypothesis, our results show that keratinocytes undergoing terminal differentiation release higher levels of IL-38 compared with IL-36Ra. However, IL-17, IL-22, and IL-36γ, known to inhibit the terminal keratinocyte differentiation^8,20^, have inhibitory effects on IL-38 expression, although they induce IL-36Ra antagonist. A predictive analysis of potential transcriptional factor-binding sites on IL-38 and IL-36Ra promoter sequences revealed the presence in the promoter of IL-38, but not of IL-36Ra, of elements likely binding to cAMP-responsive repressors, such as E4BP4 and ICER, and Kruppel-like factors, known to be induced by inflammatory cytokines and related to keratinocyte differentiation^35–37^. These factors could be responsible for IL-38 downregulation in keratinocytes subjected to de-differentiative processes triggered by IL-36γ, IL-17A, and IL-22. IL-38 expression varies in different disease settings, especially in disorders with dysregulated IL-1-dependent responses, such as spondylitis ankylopoetica, rheumatoid arthritis, and HS. In our study, we found that the local decline of IL-38, and the concomitant induction of IL-36γ and IL-36Ra in psoriatic epidermis, are common to other inflammatory skin diseases characterized by a prominent neutrophilic infiltrate, including not only HS, but also Sweet’s syndrome and PG, where an altered epidermal architecture has been reported^38,39^. Contrarily, a modest IL-38 expression has been found in skin affected by AD, an immune-mediated skin disease characterized by a prominent Th2 lymphocyte infiltrate and lacking of neutrophil circuits^40^, thus, supporting the involvement of the IL-36 cytokine axis in cutaneous skin disorders determined by neutrophil presence. Furthermore, we investigated the effects of IL-38 reinforcement in in vitro and in vivo models of psoriasis. In line with data reported by Boutet et al., we found that the endogenous expression of IL-38 is significantly reduced in the skin of IMQ-induced mice, whereas that of IL-36Ra is induced and correlates with IL-36γ upregulation, thus reflecting the expression pattern of these cytokines in psoriatic skin lesions. Interestingly, we demonstrate that the administration of IL-38 significantly attenuates the severity of psoriasiform phenotype induced by IMQ, by restoring the physiological proliferation and differentiation programs in keratinocytes, and decreasing the expression of VEGF-A, a cytokine involved in the altered angiogenic processes occurring in psoriasis^41^. IL-38 reduces infiltration of T cells and neutrophils in IMQ model, accordingly with a significant reduction of psoriasis-related chemokines involved in T-cell and neutrophil recruitment and activation. The ameliorative impact of IL-38 on the psoriatic clinical phenotype is also associated with the decrease of IL-36 local expression, and was comparable to that observed with IL-36Ra, whose anti-inflammatory properties have been recently demonstrated also in an ex vivo model of human psoriasis^16^. Recently, Palomo et al.^42^ demonstrated that the depletion of IL-38, differently from IL-36Ra, does not affect the severity of the psoriasiform reactions in IMQ-treated mice. These results are not surprising if we consider that endogenous IL-38 is per se absent in skin of this mouse model, whereas IL-36Ra is upregulated. In our opinion, IL-38 reinforcement could be the better experimental approach to evaluate its function in inflammatory skin conditions, and the ameliorative effects of the IL-38 administration in IMQ model confirm our thesis.

In our study, contrarily to what observed in vivo in IMQ-induced model, the anti-inflammatory effects of IL-38 and IL-36Ra are not entirely comparable in vitro, being IL-36Ra able to inhibit, at the same doses, the inflammatory responses more efficiently than IL-38. This discrepancy can be explained considering that we use a full-length IL-38, whereas IL-36Ra was employed in a more active, N-terminally cleaved, form^7,43^. As two N-terminally cleaved forms of IL-38 with an increased inhibitory activity have been identified in tumor cells^44^, we hypothesize that IL-38 is processed in our in vivo experimental system, likely owing to extracellular neutrophil proteases abundantly released in the IMQ-treated skin^31,45,46^. Finally, the absence of a dose–response effect of IL-38 (as well as of IL-36Ra) on in vitro expression of inflammatory mediators support the hypothesis of Dinarello et al. that IL-38 and IL-36Ra do not behave as classic receptor antagonists^5^. Upon binding to IL-36R, IL-38, and IL-36Ra might recruit an inhibitory co-receptor at low concentrations, but a signaling co-receptor at higher concentrations, similarly to what observed for IL-37^5^. In summary, IL-38 has strong anti-inflammatory effects on IL-36-induced responses in the psoriatic skin context, making it an attractive cytokine to explore also in other Th17-related diseases characterized by dysregulated IL-1-driven responses. Clinical trials with an IL-36R blocking antibody (ANB019) are ongoing in generalized pustular psoriasis and palmoplantar pustular psoriasis. These studies represent the first attempt to block inflammatory responses executed by epidermal keratinocytes, the primary source of IL-36 cytokines. However, the reinforcement of IL-38 could represent an alternative therapeutic strategy for inhibiting IL-36-induced responses in psoriasis, although understanding the functional biology of IL-38 in skin context will be needed to reveal its role in new mechanisms of disease and to evaluate the potential impact of its manipulation in psoriasis treatment.

## Materials and methods

### Human subjects

Twenty-five patients with mild-to-severe chronic plaque psoriasis (4 ≤ PASI ≥ 45), and 25 healthy subjects undergoing plastic surgery were enrolled in this study (age range 18–70 years). Psoriatic patients had similar characteristics, including age of onset and disease duration and, after an induction phase, they received subcutaneous injections of 300 mg Secukinumab once a week (Cosentyx, Novartis).

Biopsies were taken from skin plaques at sites overlapping LS before treatment (*T* = 0) and the adjacent Pre-LS. Biopsies at LS sites after 8-week treatment (*T* = 8) and at NLS, 3 cm distant from the evolving plaques, were also taken from the same psoriatic patients. In parallel, skin biopsies were also taken by healthy volunteers undergoing plastic surgery, and from lesional skin areas of patients with HS, SS, PG, and AD. Among individuals, 8 were selected for immunohistochemical and 10 for RT-PCR studies, whereas all 25 were analyzed for IL-36 serum quantification. This study was approved by the Ethical Committee of the IDI-IRCCS Hospital, Rome (registration no.: IDI-IMM-IL36pso) and performed accordingly to the Declaration of Helsinki. Informed consent was signed by all study subjects.

### Cell cultures and treatments

Normal human keratinocytes were established from sun-protected skin of healthy individuals (*n* = 4) and cultured as previously reported^47^. Cells were plated at 5000/cm^2^ and maintained to 4-d post confluency. High-confluence cultures were stimulated with recombinant human IL-22 (50 ng/ml), IFN-γ (200 U/ml), IL-17A (50 ng/ml), TNF-α (50 ng/ml), or IL-36γ (Ser18-Asp169; 50 ng/ml) (R&D Systems, Minneapolis, MN, USA) in keratinocyte basal medium (Clonetics).

HDMEC previously isolated from foreskin of three different donors^48^ were retrieved from the Cell Bank of the Laboratory of Experimental Immunology of IDI-IRCCS and stimulated with TNF-α (10 ng/ml), IL-17A (50 ng/ml), IFN-γ (200 U/ml), or IL-36γ (50 ng/ml) in endothelial basal medium (EBM, Lonza) containing 2% fetal bovine serum (starvation medium). When necessary, both cell types were treated with recombinant human IL-38 or IL-36Ra (mature form Val2-Asp155; R&D Systems) concomitantly to cytokine stimulation.

### Recombinant expression and purification of IL-38

A single Gibson block (gblock, IDT technologies) was produced encoding for an N-terminal 6xHis tag, a thrombin cleavage site, and full-length IL-38 (152 residues). This gblock was PCR amplified for insertion into pET21 using a single NdeI site and subsequently expressed in BL21/DE3 cells in loria broth supplemented with 100 mg/ml ampicillin. Cells were grown until an optical density (600 nm) of ~ 0.6 and induced for 3–4 h prior to harvesting. The full-length protein was also frown in M9 minimal media to obtain 15N-labeled protein in order to confirm that IL-38 was well-folded (data not shown).

For IL-38 purification, cell pellets were sonicated on ice in lysis buffer (50 mM Na_2_HPO_4_, pH 7.5, 500 mM NaCl, 10 mM imidazole), centrifuged to remove cellular debris, and applied to a Ni-affinity column (GE Healthcare Life Sciences. IL-38 was eluted using lysis buffer supplemented with 400 mM imidazole. Fractions comprising IL-38 were dialyzed against “Q buffer” (50 mM tris, pH 8.0, 50 mM NaCl, 10 mM imidazole, 1 mM dithiothreitol), applied to a Q-sepharose fast flow column (GE Healthcare Life Sciences). Finally, fractions comprising IL-38 were concentrated, cleaved with thrombin at room temperature overnight to remove the 6xHis tag, and applied to a Superdex-75 (GE Healthcare Life Sciences) in “final buffer” (10 mM HEPES, pH 70, 150 mM NaCl). IL-38 was concentrated and stored at − 80 °C until further use.

### IMQ-induced psoriasiform model

Eight-week-old female BALB/cJ mice (Harlan Laboratories, San Pietro al Natisone, Italy) treated for 5 consecutive days with 5% (62.5 mg) IMQ (ALDARA cream, Meda AB, Solna, Sweden)^49^ received daily subcutaneous injections (1 μg/mouse) of human recombinant full-length IL-38 (*n* = 8), Val2-Asp155 IL-36Ra (*n* = 8), or control vehicle (PBS) (*n* = 8), starting on day 0 of IMQ administration. A mice group (*n* = 3), not receiving IMQ, was used as negative control. On day 5, full thickness skin biopsies of the treated area were collected with an eight-mm biopsy puncher. Skin was either snap frozen in liquid N_2_ for total RNA preparation, or fixed in neutral buffered formalin (Sigma-Aldrich, St. Louis, MO, USA) for histopathological analysis. The whole experiment was repeated twice with similar results. All mouse procedures were carried out in accordance with institutional standard guidelines. The experimental design has been authorized by the Italian Health Minister (protocol No. SA-IDI-13-CA-1).

### Immunohistochemistry

Paraffin-embedded sections were obtained from biopsies of psoriatic skin including LS at baseline (*T* = 0) and after 8-week Cosentyx treatment, Pre-LS and NLS zones of evolving plaques, as well as of healthy skin. Paraffin-embedded sections obtained from patients affected by HS, AD, SS, or PG syndromes were retrieved by Histopathology Unit at IDI-IRCCS hospital.

Immunohistochemistry analyses were performed with primary antibodies against IL-38 (InVitrogen, Carlsbad, CA, USA), IL-36Ra (AbCam, Cambridge, UK) and IL-36γ (AbCam). Secondary biotinylated mAbs and staining kits (Vector Laboratories, Burlinagame, CA, USA) were used to develop IL-38 and IL-36Ra immunoreactivities, whereas biotinylated secondary Ab and staining kit from Scytek (Logan, Utah, USA) was used for IL-36γ detection.

Paraffin-embedded sections were obtained from murine skin tissues and analyzed for epidermal and scale thickness, as well as cell infiltrate number. Average epidermal and scale thickness was quantified by a researcher blind to the experimental groups who took five measurements per three sections for each mouse. Cells infiltrating dermis were also counted in three skin sections for each mouse. Immunohistochemistry analyses were performed with primary Abs against CD3 (Dako, Glostruk, Denmark), CD11c (Dako, Glostruk, Denmark), Ly6G (BD Biosciences, Franklin Lakes, NJ USA), Ki67 (Novocastra, Newcastle upon Tyne, UK), KRT10 (Covance, Princeton, NJ, USA), VEGF-A (Santa Cruz Biotechnology) and immunoreactivities developed with secondary biotinylated mAbs and staining kits (Vector Laboratories, Burlingame, CA, USA). All sections were counterstained with Mayer’s Hematoxylin and eosin and positivity was evaluated in five adjacent fields at a magnification × 200.

A semiquantitative, four-stage scoring system was applied, ranging from negative immunoreactivity (0) to strong immunoreactivity (4+) for IL-36 cytokines in human skin, as well as for KRT10 and VEGF-A in murine skin. IL-36 cytokine-positive cells were counted blindly by two observers with an eyepiece graticule at a magnification of × 200. For each skin specimen, two sections were analyzed for each staining and positive cells were evaluated in five adjacent fields.

### RNA isolation and real-time RT-PCR

Total RNA from keratinocytes and endothelial cells was extracted using the TRIzol reagent (InVitrogen), whereas total RNA from human and mouse skin biopsies by RNeasy Lipid Tissue Kit (Qiagen, Chatsworth, CA, USA). mRNA was reverse-transcribed into complementary DNA and analyzed by real-time PCR. GAPDH or β-2 microglobulin were used as housekeeping genes for human and murine mRNA, respectively. Primer pairs used in PCR reactions are listed in the table reported in Supplementary Table S1. Fluorescence intensity was analyzed by the ABI PRISM SDS 7000 PCR Instrument (Applied Biosystems, Branchburg, NJ, USA), using SYBR Green PCR reagents or Taqman PCR Master Mix. The values obtained from triplicate experiments were averaged, and data are presented as means ± SD.

### Cytokine quantification by ELISA

Concentrations of IL-38, IL-36Ra, and IL-36γ were measured in serum obtained by venous blood of patients enrolled in this study, as well as in cell-free supernatants from untreated or cytokine-treated keratinocyte and endothelial cell cultures, using specific ELISA kits (BD Pharmingen). The plates were analyzed in an ELISA reader mod.3550 UV (Bio-Rad Hercules, CA, USA).

### Western blotting analysis

Total protein extracts by cell cultures and immunoblotting were performed as previously reported^36^. The Abs employed for the study were as follows: anti-phospho-Erk1/2 (E-4), anti-Erk1/2 (C16), anti-β-actin (C-11), all provided by Santa Cruz Biotechnology (Santa Cruz, CA, USA). Anti-phospho-P65, anti-P65, anti-phospho-P38, and ant-P38 were from Cell Signaling Technology (Denvers, MA, USA), whereas anti-KRT1, anti-KRT10, and anti-loricrin were from Covance (Emeryville, CA, USA). Anti-ΔNp63 Ab was provided by Santa Cruz Biotechnology. Filters were properly developed with anti-mouse, anti-goat, or anti-rabbit Ig Abs conjugated to horseradish peroxidase using the ECL-plus detection system (Amersham, Dubendorf, Switzerland), or, otherwise, the SuperSignal West Femto kit (Pierce, Rockford, IL, USA). Immunoblots were subjected to densitometry using an Imaging Densitometer model GS-670 (Bio-Rad) supported by the Molecular Analyst software, and band intensities were evaluated in three independent experiments. Data are expressed as densitometric intensity (DI) ± SD.

### HDMEC proliferation

In total, 6 × 10^4^ HDMEC were seeded in 12-well plates in endothelial growth medium in triplicate for each condition. 1 day after, the cells were starved in EBM and treated with IL-36γ alone or in presence of recombinant IL-38 or IL-36Ra and cultured for 24 and 48 h. The number of viable cells was determined by a trypan blue exclusion test.

### Flow cytometry analysis

Keratinocyte and HDMEC membrane expression of ICAM-1 was evaluated using allophycocyanin (APC)-conjugated anti-CD54 monoclonal Ab (clone 84H10; Immunotech, Marseille, France), whereas VCAM expression on HDMEC membrane was detected by using APC-conjugated anti-human VCAM-1 (clone 51-10C9, BD Pharmingen). Cells were analyzed by a FACScan equipped with Cell Quest software (Becton Dickinson, Mountain View, CA, USA).

### Statistical analysis

Differences between groups were evaluated by the Mann–Whitney *U* or unpaired Student‘s *t* test as specified in the figure legends using GraphPad prism Software (La Jolla, CA, USA). Spearman’s test was used to correlation analysis.

## Electronic supplementary material

Figure S1

Figure S2

Table S1

Supplementary figure legends
