# Supplementary figures and images for: IL-38 has an anti-inflammatory action in psoriasis and its expression correlates with disease severity and therapeutic response to anti-IL-17A treatment
Source: Cell Death Dis. 2018 Oct 30;9(11):1104. doi: 10.1038/s41419-018-1143-3 (PMC6207563; doi:10.1038/s41419-018-1143-3)

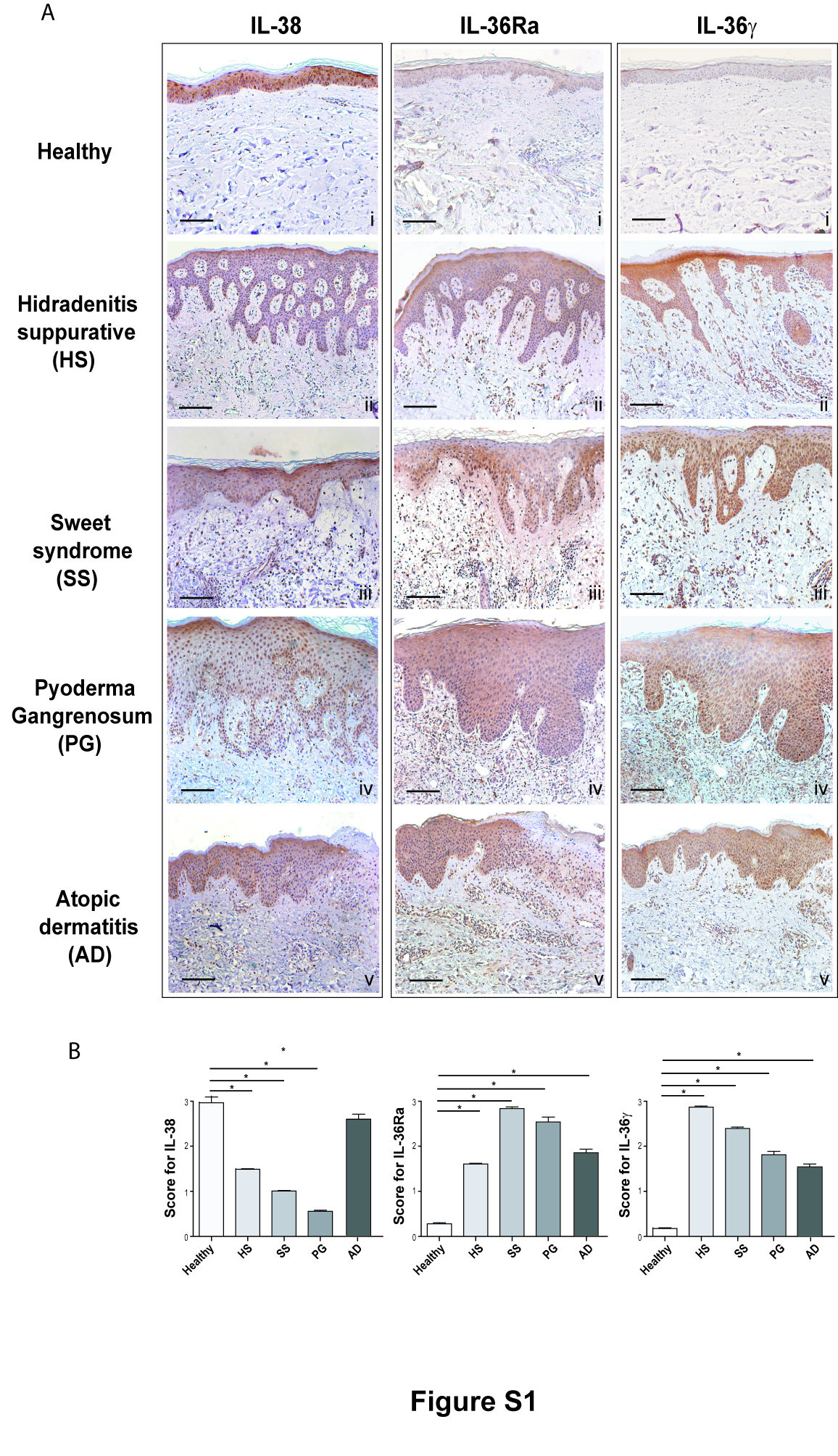

Supplement: Supplementary file 1 — Figure S1 [file 41419_2018_1143_MOESM1_ESM.tif]

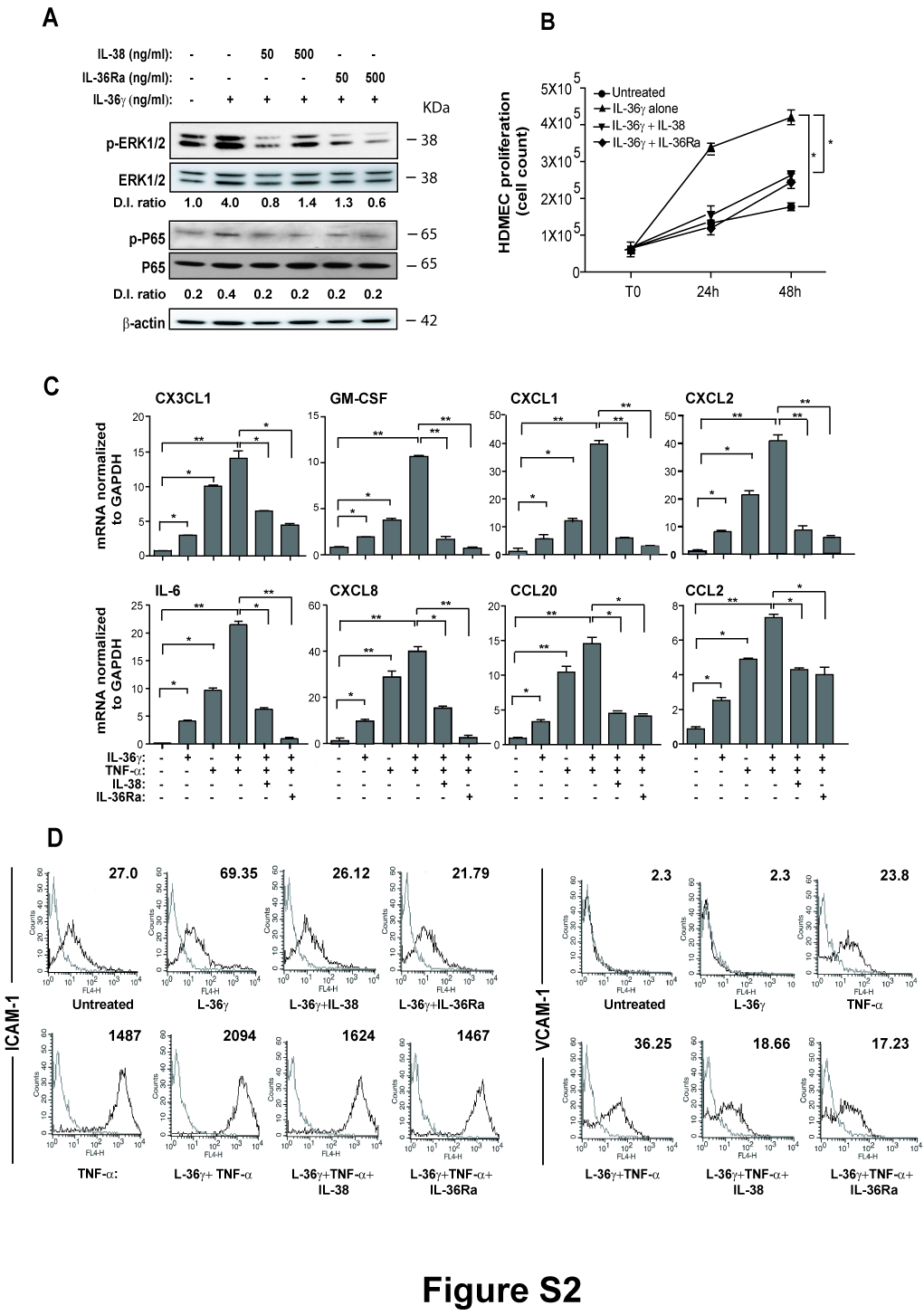

Supplement: Supplementary file 2 — Figure S2 [file 41419_2018_1143_MOESM2_ESM.tif]
