# Supplementary material for: IL-38 has an anti-inflammatory action in psoriasis and its expression correlates with disease severity and therapeutic response to anti-IL-17A treatment
Source: Cell Death Dis. 2018 Oct 30;9(11):1104. doi: 10.1038/s41419-018-1143-3 (PMC6207563; doi:10.1038/s41419-018-1143-3)
Supplement: Supplementary file 3 — Table S1 [file 41419_2018_1143_MOESM3_ESM.docx]

| Human |  | Sequence |
| --- | --- | --- |
| IL-38 | Fw | 5’-AAG GTC CCC ATT TTC CTG GG- 3’ |
|  | Rv | 5’-CTC AAT GTT CAC ATC CTC CAG C- 3’ |
| IL-36Ra | Fw | 5’-GTG CTT CCG AAT GAA GGA CT- 3’ |
|  | Rv | 5’-GAC CAC GCT GAT CTC TTC AC- 3’ |
| IL-36γ | Fw | 5’-GCC GTC TAT CAA TCA ATG TGT AA- 3’ |
|  | Rv | 5’-GAA CTG CCA CAA GGT TCT GA- 3’ |
| CXCL8 | Fw | 5’-CCC CTA AGA GCA GTA ACA GTT CCT- 3’ |
|  | Rv | 5’-GGT GAA GAT AAG CCA GCC AATC- 3’ |
| CCL20 | Fw | 5’-GTG CTG CTA CTC CAC CTC TG- 3’ |
|  | Rv | 5’-TGT ATC CAA GAC AGC AGT CAAA- 3’ |
| VEGF-A | Fw | 5’-TCACCAAGGCCAGCACATAG-3’ |
|  | Rv | 5’-TCGGCTTGTCACATCTGCAT-3’ |
| IL-6 | Fw | 5’-GGCACTGGCAGAAAACAACC-3’ |
|  | Rv | 5’- CACCAGGCAAGTCTCCTCAT-3’ |
| HBD-2 | Fw | 5’-TCC TCT TCT CGT TCC TCT TCA TATT- 3’ |
|  | Rv | 5’-TTA AGG CAG GTA ACA GGA TCGC- 3’ |
| HBD-2 probe | Fw | 5’-ACC ACC AAA AAC ACC TGG AAG AGG CA- 3' |
| LL-37 | Fw | 5’-TTT TGC GGA ATC TTG TAC CCA- 3’ |
|  | Rv | 5’-TCT CAG AGC CCA GAA GCC TG- 3’ |
| CX3CL1 | Fw | 5’- TCACGTGCAGCAAGATGACA-3’ |
|  | Rv | 5’- TCTCCAAGATGATTGCGCGT-3’ |
| GM-CSF | Fw | 5’-GCGTCTCCTGAACCTGAGTAG-3’ |
|  | Rv | 5’-TCGGCTCCTGGAGGTCAAAC-3’ |
| CXCL1 | Fw | 5’-CCTCAATCCTGCATCCC-3’ |
|  | Rv | 5’-AGTTGGATTTGTCACTGT-3’ |
| CXCL2 | Fw | 5’- GAAAGCTTGTCTCAACCCCG-3’ |
|  | Rv | 5’- TGGTCAGTTGGATTTGCCATTTT-3’ |
| CCL2 | Fw | 5’-CAC CAG CAG CAA GTG TCCC- 3’ |
|  | Rv | 5’-CCA TGG AAT CCT GAA CCC AC- 3’ |
| GAPDH | Fw | 5’-TGG ACC TGA CCT GCC GTC TA - 3’ |
|  | Rv | 5’-CCC TGT TGC TGT AGC CAA ATT C - 3’ |

Supplementary table S1.

**Legend to table**: List of primer sequences used for Real-time PCR analysis on human samples.
