# Supplementary material for: IL-38 has an anti-inflammatory action in psoriasis and its expression correlates with disease severity and therapeutic response to anti-IL-17A treatment
Source: Cell Death Dis. 2018 Oct 30;9(11):1104. doi: 10.1038/s41419-018-1143-3 (PMC6207563; doi:10.1038/s41419-018-1143-3)
Supplement: Supplementary file 4 — Supplementary figure legends [file 41419_2018_1143_MOESM4_ESM.docx]

**Supplementary Figure legend**

**Fig. S1** ***In vivo* expression of IL-38, IL-36Ra and IL-36γ in skin inflammatory diseases. a** Immunohistochemistry for IL-38, IL-36Ra and IL-36γ (all stained in red-brown) was performed on parafﬁn-embedded sections obtained from skin of healthy volunteers (n = 6) (panels i) and of patients affected by HS (panels ii), SS (panels iii), PG (panels iv) and AD (panels v) (n = 8 for each syndrome). Sections were counterstained with Mayer’s H&E. One out of eight representative stainings of skin biopsies are shown. Bars, 100 μm. With the exception of AD, a relevant percentage of IL-36Ra^+^ and IL-36γ^+^ cells with a neutrophil-like morphology was observed in immune cells (mean of IL-36Ra^+^ immune cells: 30 ± 2.4% in HS, 32 ± 2.1% in SS and 45 ± 3.1% in PG; IL-36γ^+^ cells: 40 ± 2.8% in HS, 35 ± 2.1% in SS and 50 ± 3.2% in SS).

**b** Graphs show the mean of four-stage score values for IL-38, IL-36Ra and IL-36γ ± SD per three different fields of all six sections. **p* ≤ 0.05 compared to healthy skin, as assessed by Mann-Whitney *U*-test.

**Fig. S2 IL-38 inhibits IL-36γ-induced ERK and P65 phosphorylation, as well as it counteracts the HDMEC proliferation and expression of pro-inflammatory molecules**. **a** Protein extracts were obtained from HDMEC cultures stimulated or not with IL-36γ in presence or absence of increasing doses of IL-38 or IL-36Ra, and subjected to WB analysis to detect ERK1/2 and P65 phosphorylation. Filters were probed with anti-ERK1/2 and -P65 Abs. D. I. ratio indicates the densitometric intensity of the indicated phosphorylated/unphosphorylated proteins shown in one representative of three different WB. **b** HDMECs were cultured with IL-36γ alone and in presence of IL-38 or IL-36Ra (both at 500 ng/ml) and the proliferation was evaluated after 24 and 48 h of culture by a trypan blue exclusion test. In (**c**) and (**d**) HDMEC were stimulated with IL-36γ and TNF-α alone or in combination, in presence or not of IL-38 or IL-36Ra (both at 500 ng/ml). **c** mRNA levels of CX3CL1, GM-CSF, CXCL1, CXCL2, IL-6, CXCL8, CCL20 and CCL2 were detected by real-time PCR in HDMEC stimulated for 6 hours. GAPDH levels were detected for normalization. (**d**) The expression of ICAM-1 and VCAM-1 was evaluated by ﬂow cytometry analysis in HDMEC stimulated with cytokines for 24h. Data are shown as mean ﬂuorescence intensity. All data shown are the mean values of three different experiments performed. **p* ≤ 0.05; *p*** ≤ 0.01 compared to untreated or cytokine-treated cultures, as assessed by Mann-Whitney *U*-test.
